# Chromatin organization modulates the origin of heritable structural variations in human genome

**DOI:** 10.1093/nar/gkz103

**Published:** 2019-02-18

**Authors:** Tanmoy Roychowdhury, Alexej Abyzov

**Affiliations:** Mayo Clinic, Department of Health Sciences Research, Center for Individualized Medicine, Rochester, MN 55905, USA

## Abstract

Structural variations (SVs) in the human genome originate from different mechanisms related to DNA repair, replication errors, and retrotransposition. Our analyses of 26 927 SVs from the 1000 Genomes Project revealed differential distributions and consequences of SVs of different origin, e.g. deletions from non-allelic homologous recombination (NAHR) are more prone to disrupt chromatin organization while processed pseudogenes can create accessible chromatin. Spontaneous double stranded breaks (DSBs) are the best predictor of enrichment of NAHR deletions in open chromatin. This evidence, along with strong physical interaction of NAHR breakpoints belonging to the same deletion suggests that majority of NAHR deletions are non-meiotic i.e. originate from errors during homology directed repair (HDR) of spontaneous DSBs. In turn, the origin of the spontaneous DSBs is associated with transcription factor binding in accessible chromatin revealing the vulnerability of functional, open chromatin. The chromatin itself is enriched with repeats, particularly fixed Alu elements that provide the homology required to maintain stability via HDR. Through co-localization of fixed Alus and NAHR deletions in open chromatin we hypothesize that old Alu expansion had a stabilizing role on the human genome.

## INTRODUCTION

Structural variations (SVs) are defined as large DNA variants (e.g. insertions, deletions, duplication) that are longer than 50 bp (1–3). Although, number of events is smaller, these SVs represent a larger number of variable bases among personal genomes than SNVs (4). Understanding the origin of these SVs is important due to their frequent association with different diseases (4–8). Advances in next-generation sequencing and computational techniques have enabled precise identification of SVs (at base-pair resolution) that can be further used to identify mechanisms of SV origin (2). Broadly, SVs are classified as repair-related, retrotransposition-related or repeat polymorphisms; these groups can be further subdivided into multiple categories. Because of etiologic differences, each class of SV is expected to be associated with different mechanistic biases that might affect phenotypes in varying ways (4). On the other hand, advances in epigenomic studies have enabled deeper understanding and annotation of chromatin structure and function of different regions of the genome. Hi-C experiments have shown that there are six compartments in the genome, each representing different patterns of long-range inter-chromosomal interaction (9). Two of these compartments (A1 and A2) correspond to open chromatin, whereas the remaining four compartments (B1, B2, B3 and B4) represent closed chromatin. Overall, A type compartments are usually enriched with DNase I hypersensitive sites, expressed genes, activating histone marks and early replication time. In contrast, B type compartments are associated with repressive histone marks, late replication, nuclear lamina and nucleolus-associated domains. While these compartments provide a lower resolution annotation of epigenomic properties, histone marks, replication timing, DNase hypersensitive sites, nucleosome positioning define genome organization in higher resolution. In this study, we analyzed germline SVs of different origin in the context of high and low resolution epigenomic properties to understand their etiologic basis and thereby gain insight into their biases in genomic distribution, selection and functional consequences.

## MATERIALS AND METHODS

### Datasets

SV breakpoint coordinates and their possible mechanism of origin were obtained from phase 3 of the 1000 Genomes Project (1). These co-ordinates are according to the hg19 release. Non-homologous deletions were further classified into two categories based on micro-homology and micro-insertion. Non-homologous deletions with micro-homology greater than 2 bp or micro-insertion longer than 10 bp is evidence of replication related errors (NHrepl), whereas absence of these suggests non-homologous end joining (NHEJ). AluY and AluS elements were obtained from the UCSC Genome Browser, and Alu elements (280–350 bp) without any overlap with the SV set were selected as non-variable elements. Coordinates of spontaneous double stranded break (DSB) peaks were obtained from GEO with accession number GSE78172 (BREAK.common_n1_n2.default_nomodel.bed) and meiotic DSB peaks from GEO with accession number GSE59836 (GSE59836_Peak_data_Supplementary_File_1.txt). Gender specific recombination rates were obtained from http://www.decode.com/addendum. Processed pseudogenes were obtained from human table of http://pseudogene.org; those with >95% sequence identity to the parent gene and length>250 bp were used for analysis. A pseudogene was classified as having a length close to nucleosome periodicity if, after dividing the length by 150, the remainder was 0–50 or 100–150. DNase I hypersensitive peaks and nucleosome signal for NA12878 were obtained from ENCODE. Non-allelic homologous recombination (NAHR) mediated deletion breakpoints in cancers were obtained from Zhuang *et al.* (10). This study classified NAHR deletions from 97 cancer genomes to be of somatic or germline origin. The merged transcription factor binding site set was obtained from Sabarinathan *et al.* (11).

### Simulated NAHR

Random sequences (100 bp) were obtained from the reference genome and mapped to the whole genome using blastn to find paired homologous sequences. Sequences that could be mapped within 1 MB of the origin (but not less than 100 bp) were considered for the next step. Next, only alignments with same orientation, length >50 bp, and sequence identity >85% were considered. If, the original sequence had >1 such alignment, only 1 target location was chosen randomly. These thresholds were chosen according to simulations performed in supplementary Figure of Lam *et al.* (12). The locations of the original and target sequences were considered as two breakpoints of a simulated NAHR event. This set was termed as simulated homologous breakpoints. Next, probability distribution function of length was calculated from real NAHR deletions. For each simulated breakpoint, a random fraction between 0 and 1 was chosen. That specific breakpoint was accepted for the next step if it could be plotted in the area under the curve of the probability distribution function (x-axis, length; y-axis, random number). From real NAHR deletions, we obtained the proportions of breakpoints for each compartment. Simulated NAHR breakpoints from each compartment were randomly chosen in the same proportions to obtain the set of advanced simulated NAHR.

### Background distribution

As a starting point, the whole genome was imagined to be circular, with sequentially arranged chromosomes (i.e. 1, 2, 3, …, *Y*). To create a circular permutation, a random number from 1 to the length of the human genome was generated and added to the original coordinate of each SV. Thus, we obtained a new location for each SV (equally shifted from the original locations), with their lengths unchanged from the original. This procedure was repeated 1000 times to generate 1000 sets of circularly permuted SVs. Calculation of any property generated a background distribution from 1000 instances.

### Analysis of biased SV distribution

Compartment, topologically associated domain, and loop annotation of NA12878 were obtained from GEO with accession number GSE63525. Compartment information from NA12878 consisted of 4064 bins, each annotated with 1 of the 6 compartments. Each breakpoint was assigned to a compartment by identifying the bin in which the breakpoint resided. For each mechanism, the fraction of events residing in each of six compartments was calculated. Similarly, these fractions were calculated from the 1000 instances of background SV distributions, which enabled calculation of means and standard deviations that were used to calculate *Z* scores for each mechanism and compartment. Annotation of 18 550 topologically associated domain boundaries and 18 898 loop anchors were used to identify SVs that encompassed these locations. Similarly, *Z* scores were calculated compared to background events encompassing these locations.

Replication time was obtained from Koren *et al.* (13). Each base in the genome was annotated with a replication time ranging from –2.10 to 2.77, with positive values representing early replication and negative values representing late replication. The replication time for each breakpoint was calculated by averaging the replication time of 1000 bases (500 on each side around the breakpoint). Mechanisms were clustered based on *P* values obtained by Kolmogorov–Smirnov tests performed on the distribution of replication time around the breakpoint corresponding to each mechanism.

A 15-state model, trained using five histone marks from 127 epigenomes jointly, was used for the analysis based on chromHMM. ChromHMM segmentation of NA12878 was obtained from epigenome Roadmap (https://egg2.wustl.edu/roadmap/web_portal/chr_state_learning.html). We followed the same naming convention for these 15 states as the original data. Z scores for breakpoints were calculated in the same way as described above for compartments.

### Regression analysis

We calculated the fraction of NAHR breakpoints (*Y*), simulated homologous breakpoints (*X*_1_), spontaneous (*X*_2_) and meiotic DSB peaks (*X*_3_) in each of the 4064 compartment bins. Linear regression was performed by using the lm() function in *R*, with fraction of NAHR breakpoints as the outcome variable and the remaining three as predictor variables, both separately (*Y* ∼ *X*_*n*_) and together (*Y* ∼ *X*_1_ + *X*_2_ + *X*_3_). We used the following fitted equation, *Y* = −6.440 × 10^−5^ + 0.29*X*_1_ + 0.69*X*_2_ + 0.27*X*_3_ to calculate predicted NAHR fraction in each bin. Random forest regression was performed by R package ‘randomForest’.

### Hi-C interaction analysis

For this analysis, we used intra-chromosomal contact matrices of NA12878 (1 kb resolution) that were obtained from GEO (accession number GSE63525). We used only the contact matrices that were constructed by using read pairs that mapped to the genome with MAPQ ≥30. Raw contact matrices were transformed into Knight–Ruiz (KR) normalized obs/exp matrices. To calculate compartment specific interaction profiles, each interaction in KR normalized obs/exp matrices was assigned a compartment if both interacting regions were in the same compartment. From these, we calculated compartment specific average interactions for each distance. We used only those SVs that were longer than 5kb. Two ends of an event were assigned to two separate 1 kb bins, and the corresponding Hi-C interaction value was obtained from KR normalized obs/exp matrices. These values were further normalized by the compartment and distance specific average interaction values.

### Peak aggregation analysis

For peak aggregation analysis of DNase sites, spontaneous and meiotic DSBs around breakpoints, for each 100 bp, fraction of bases that overlap peaks were calculated for 2.5 kb upstream and downstream. Aggregated signal from all sites for each mechanism was normalized by number of breakpoints in that class. Similar analysis was performed for aggregation of spontaneous DSB around DNase and transcription factor binding sites, with two exceptions. First, the mid-points of regulatory sites were used instead of breakpoints and second, 10 bp bins were used for peak overlap instead of 100 bp bins. In case of nucleosome occupancy analysis, for repair related deletions, the signal on the left and right sides of each breakpoint of all SVs were considered for aggregation. However, for retrotransposons and pseudogenes, signals were aggregated for upstream of the element start site and downstream of the element end site to avoid bias related to length conservation of retrotransposable elements. For nucleosome occupancy, each point represented 10 bp and was calculated for 1 kb upstream and downstream. Raw signal for each base in the 10 bp was added and divided by the number of bases for which data were available. Signals from each point were aggregated for all sites for each mechanism and divided by the number of sites for which any signal was available for each class. These data were further normalized for each mechanism by averaging the signal over 2 kb region around the breakpoint.

### Estimating the fraction of NAHR deletions related to meiotic DSBs

The aggregated signal of meiotic DSBs around NAHR breakpoints is apparently below than that around DBSs themselves but is higher than asymptotic background and that of around NHEJ and NHrepl (Figure 3E). Moreover, the shape of the signal is similar to that of meiotic DSBs themselves. We thus assumed that the signal was sum of two signals: the one for NAHR deletions at meiotic DSBs and the other one for NAHR deletions that are unrelated to meiotic DSBs, i.e. HDR related NAHR. We denote the fraction of the former as *x*, and the fraction of the latter as 1 – *x*. At breakpoints (i.e. the zero aggregation coordinate), meiotic NAHR deletions would contribute a signal of 1, while HDR related NAHR deletions would contribute only background signal, roughly estimated as 0.04 (based on signals for NHEJ and NHrepl). Contributions of these two components would sum to 0.08, the observed for all NAHR deletions at breakpoints. That is, ${f_m}x + 0.04( {1 - x} )\ {f_s} = \ 0.08$, where *f_m_* is the fraction of meiotic DSB sites captured by experiment (14), and *f_s_* is the fraction of spontaneous DSB sites captured by experiment (15). So, *x* can be estimated as $x\ = \ ( {0.08 - 0.04{f_s}} )/( {{f_m} - 0.04{f_s}} )$. The value of *x* only slightly depends on the values of *f_m_* and *f_s_*; if they are in the range [0.5,1.0], then *x* would be in the range [0.04,0.12].

## RESULTS

**Figure 1.**
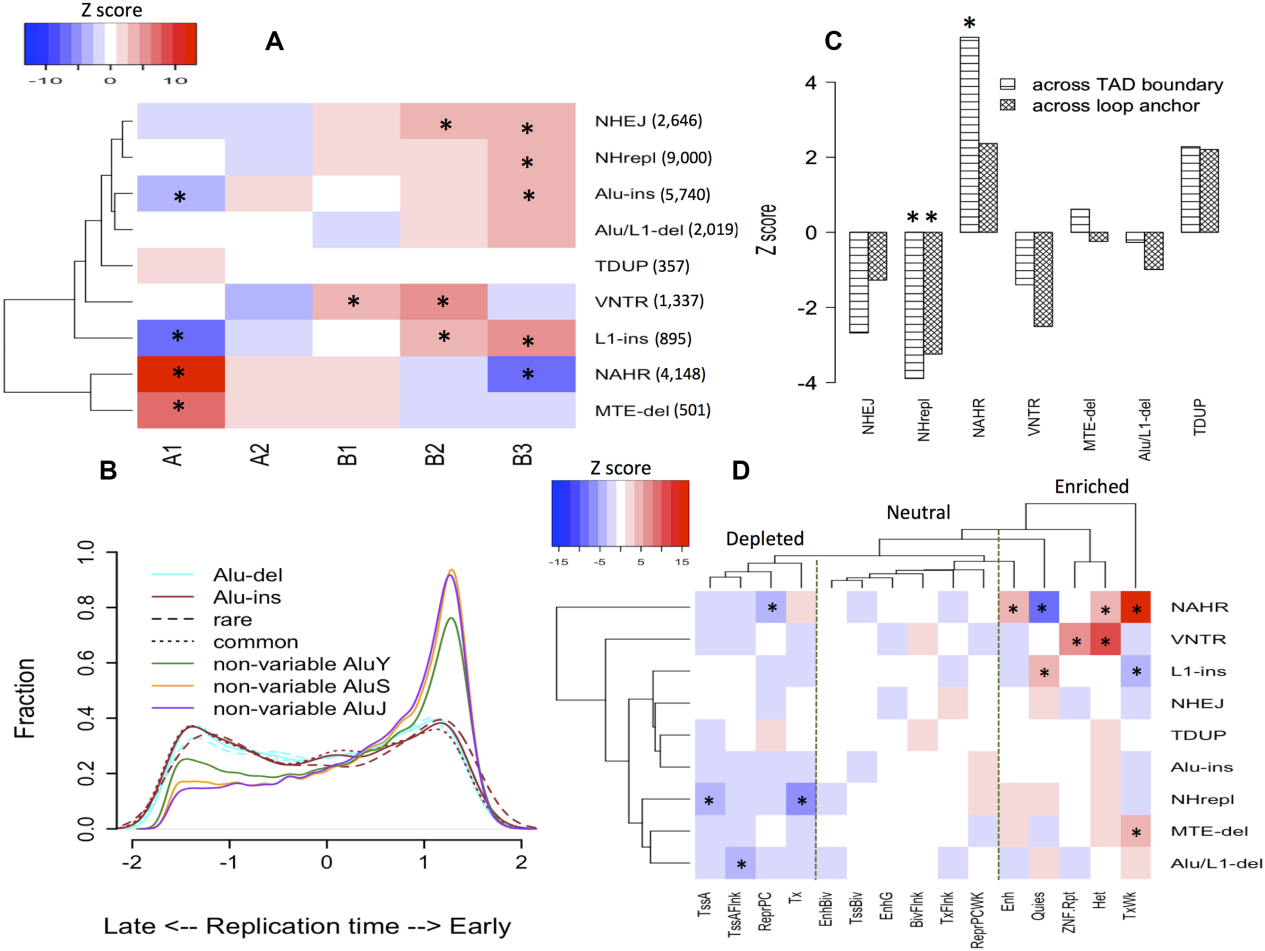
Biased distribution of different SV classes. (**A**) Clustering of SV classes based on their enrichments by Z-score in 5 compartments, identified from Hi-C studies. Two major clusters can be observed. (**B**) Normalized distribution of replication timing for different Alus. Alu insertion and deletions were further classified as rare or common (minor allele frequency threshold, 1%). (**C**) Positive and negative *Z*-scores mean enrichment and depletion, respectively, of SVs across topologically associated domain (TAD) boundary or loop anchor. (**D**) Clustering of SV classes and functional states based on distribution of breakpoints in the states. The states were inferred from chromHMM segmentation of histone marks. Descriptions of each state are provided in supplementary Table S1. Dashed lines show three categories of states by enrichment with SV breakpoints. * represents adjusted *P*-value < 0.05 (*Z*-test with Bonferroni correction). Abbreviations: Variable number of tandem repeats (VNTR), Tandem duplications (TDUP), Deletions with multiple transposable elements (MTE-del).

For our analysis, we used breakpoints of 26 927 SVs from phase 3 of the 1000 Genomes Project (1). Aided by the Breakseq tool (2,12), we classified SVs into different categories: non-allelic homologous recombination (NAHR) deletion, non-homologous deletion, single transposable element detected as a deletion (Alu/L1 deletion), deletion with multiple transposable elements, retrotransposon insertion (Alu, L1 and SVA), tandem duplication, and variable number of tandem repeats (Figure 1A and Supplementary Figure S1). We did not use SVA insertions due to smaller number of events in that category (284 for SVA compared with 5740 for Alu and 895 for L1). NAHR events likely reflect errors that occurred during directed recombination in meiosis or during homology-directed repair (HDR) of double-stranded breaks (DSBs). While an allelic HDR is unlikely to create an SV, a non-allelic HDR either lead to error-free gene conversion or deletion (16,17). Non-allelic HDR is the only choice during certain germ cell stages because of the unavailability of sister chromatid and homologous chromosome (the sister chromatid is absent in most parts of the cell cycle and the homologous chromosome is absent in haploid stages). Additionally, Hi-C data showed that homologous chromosomes occupy distinct territories in nuclear genome organization (18). Non-allelic HDR can also be preferred, even in presence of allelic templates, as had been shown in yeast (19). Non-homologous events likely arise from replication errors or errors during non-homologous end joining (NHEJ). These errors confer distinct breakpoint features in the generated deletions (20), and we, accordingly, sub-classified non-homologous deletions as NHrepl or NHEJ. For a fraction of non-homologous deletions, the distinction between these two subtypes was not obvious, and these were excluded from the analysis. Alu/L1 deletions likely originated as ancestral retrotransposon insertions of Alu and L1; because they are polymorphic, they are detected as deletions relative to the reference genome. The origins of tandem duplication and deletions with multiple transposable elements are unclear, while variable number of tandem repeats likely arose during replication slippage.

### Genome wide distributions of SVs of different origins are different

We clustered the SV mechanisms based on the distribution of their breakpoints across the 5 most common compartments, which constitute 99.7% of the human genome. From the clustering, SVs were broadly classified into two groups: those overrepresented in open chromatin (A1 and A2 compartments) and those overrepresented in closed chromatin (B1, B2 and B3 compartments) (Figure 1A). NAHR events belong to the first group but interestingly were more enriched in the A1 compartment than A2. NAHR breakpoints were observed to have the strongest enrichment (among histone marks) for H3K79me2 mark (2). The level of this histone mark was much higher in A1 than in A2 (9), suggesting a role for methylated H3K79 in enrichment differences between compartments. Indeed, this suggestion is consistent with the report of in vitro binding of repair protein 53BP1 (a key enzyme in double stranded break repair pathway) to methylated H3K79 (21) and promoting high-fidelity HDR (22). In contrast to NAHR, non-homologous deletions were more prevalent in B type compartments, whereas distribution of tandem duplications in the genome seemed to be random. These differences were unlikely to be confounded by selection or differential count in SV classes (Supplementary Figure S2). Distribution of deletions with multiple transposable element deletion was similar to that of NAHR, which implies similar origin.

It was previously shown that non-homologous and NAHR deletion breakpoints have different relationships with replication timing (2,13), which we confirmed in our analysis (Supplementary Figure S3). These findings were consistent with the distribution of SVs across compartments because replication timing is correlated with compartments. Retrotransposon insertion in the genome is likely to be random, with minor preferences for A/T rich regions. Any major differences in genomic distributions are likely caused by negative/positive selection after the elements were inserted (23). We observed enrichment of L1 insertion as well as non-variable L1 in gene-poor late replicating regions (Figure 1A, Supplementary Figure S4). Contrary to the idea of Alu elements inserting in early-replication regions (24,25), we did not found such bias for Alu elements detected either as insertions or deletions, i.e. Alu/L1-deletions with length 250–450 bp (Figure. 1A,B). However, non-variable Alu elements showed strong bias for early-replicating regions, which likely indicates substantial difference in selection between variable and non-variable Alu elements. While, some of the non-variable Alu elements can still be variable (i.e. not captured by the 1000 Genomes Project), this is unlikely to result in the large bias. This is further supported by the minor difference between AluY and AluS/J. While some of non-variable AluY elements can be polymorphic, all copies of AluJ are fixed (26). As both common and rare variable Alus show the same association with replication timing (Figure 1B), the difference is unlikely to reflect any recent evolutionary event(s). This is consistent with a previous observation in inter-species comparison between human and chimp that showed shared Alus (between human and chimp) being enriched in GC-rich regions (feature of early replication/open chromatin) while that is not the case with human/chimp specific Alus (27).

Hi-C studies have revealed the existence of topologically associating domains, which are regional units of chromatin organization that are mostly conserved across tissues. Within individual domains, DNA loops act as local units of chromatin interaction (e.g., enhancer-promoter) (28). We found that NAHR deletions affect domain boundaries more frequently than would be expected by chance (*Z*-test, adjusted *P* < 0.05) (Figure 1C). On contrary, non-homologous deletions rarely disrupt such structures. Consistent with this finding, many reported cases of domain boundary disruptions (29,30,31) have been due to inversions, which, like NAHR deletions, are also frequently generated through HDR (20).

Combination of histone marks can be used to annotate the whole genome with functional states. Clustering of SV mechanisms based on the distribution of breakpoints in 15 functional states is largely consistent with what has been observed in terms of compartments (Figure 1D). These 15 states can be broadly classified in 3 categories: depleted, neutral, and enriched with SV breakpoints. Depleted are those related to active transcription and promoter sites. The breakpoint-enriched category consists of heterochromatin and weakly transcribed regions (TxWk). General enrichment of SV breakpoints in heterochromatin (independent of the mechanism of origin) is likely due to negative selection in functional regions, leading to preferential retention of SVs in inactive regions. Enrichment of breakpoints in weakly transcribed regions and depletion in transcribed regions likely reflect association of both regions with open compartments, with stronger selection forces acting on the latter. Enhancers were enriched with breakpoints, whereas promoter elements were depleted; promoters may be subject to stronger selection pressure because of the one-to-one relationship of promoters to genes (In contrast, more than one enhancer can interact with a promoter (32)). Enrichments for NAHR breakpoints with several functional annotation categories suggest that consequences of these deletions might be greater than of non-homologous deletions, even with comparatively fewer events. These results were consistent when we considered deleted regions instead of location of breakpoints (Supplementary Figure S5).

### Majority of NAHR deletions are of non-meiotic origin

**Figure 2.**
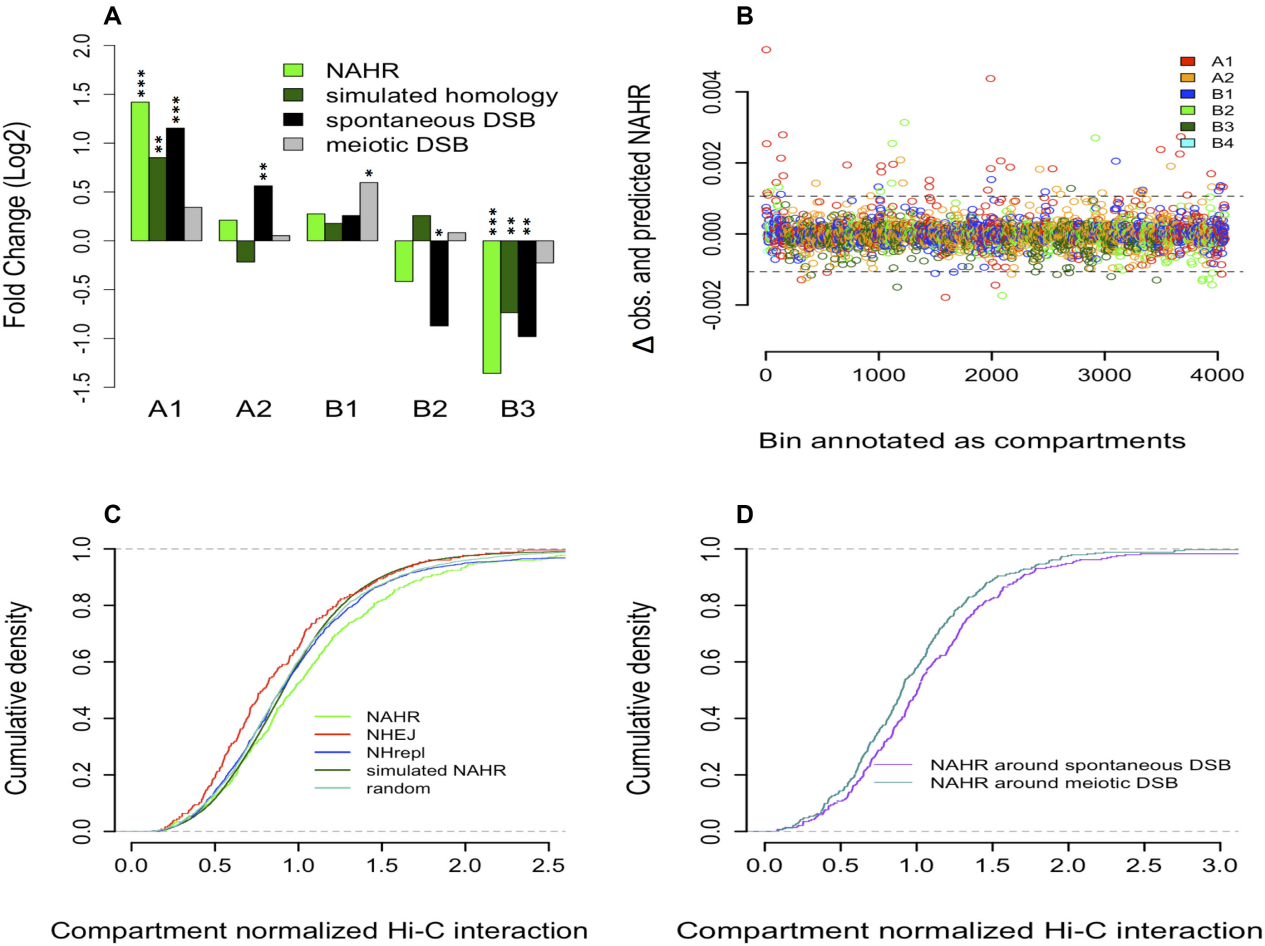
(**A**) Fold enrichment (Log_2_ scale) of real NAHR breakpoints, simulated homologous breakpoints, spontaneous and meiotic DSB in five compartments, relative to uniform distribution based on compartment lengths. *P* values were calculated using chi-square test (* indicates *P* < 0.05; **P < 0.005; ***P < 0.0005). (**B**) Differences in the number of observed and predicted NAHR events (using a multiple linear regression model) along each compartment segment. Values closer to zero imply better prediction. Dotted lines represent 3 standard deviations from the mean. Bins with values outside the dotted lines represent discordant predictions. (**C**) Cumulative density of Hi-C interaction observed between breakpoints of SVs in different classes, advance simulated NAHR breakpoints, and random points. Original interaction values were normalized based on a background distribution for each compartment. This eliminated the effect of overall stronger interactions in B compartments, facilitating an unbiased comparison (Supplementary Figure S7). (**D**) Cumulative density of normalized Hi-C interaction for NAHR breakpoints with only spontaneous DSB or only meiotic DSB within 1 kb.

To better understand genomic features associated with NAHR breakpoints, we simulated their distribution in the genome. To study dependence of NAHR on sequence homology, we generated a set of simulated homologous breakpoints with only similar repeat content to that one around breakpoints of real NAHR deletions. Although randomly chosen, the distribution of simulated homologous breakpoints in different compartments was non-random, i.e. significantly enriched in A1 but depleted in B3 (Figure 2A), portraying the biased distribution of sequence homology required for a NAHR. Furthermore, we observed a 6.1 fold enrichment (chi-square test, *P* < 0.0001) of simulated homologous breakpoints in non-variable AluS/J compared to uniform distribution.

Apart from sequence homology, a DSB is a primary requisite for NAHR events, which can be spontaneous or meiotic. Spontaneous DSB peaks represent regions where frequent breaks are caused by various mutagenic stresses (15), while the meiotic DSB peaks represent programmed DSBs induced during meiotic crossover (14). A strong bias was observed for a higher number of spontaneous DSBs in compartments with more accessible chromatin (Figure 2A). This bias was primarily driven by the density of DNase sites, as spontaneous DSBs outside DNase sites were rather uniformly distributed (Supplementary Figure S6). In contrast, meiotic DSBs were weakly associated with only the B1 compartment (Figure 2A). Consistent with this finding, gender-specific recombination rates were higher in B1 and B2 compartments (supplementary Table S2). Therefore, the overall distribution of spontaneous DSBs (and not meiotic DSBs) is most similar to that of NAHR breakpoints.

Based on compartments, the genome could be further segmented into 4064 bins (9). Using linear regression, we calculated the ability of three predictor variables (simulated homologous breakpoints, spontaneous DSBs, meiotic DSBs) to predict the fraction of NAHR events in these bins (Supplementary Figure S7). Spontaneous DSB was the best predictor of NAHR events alone (adjusted *R*^2^ = 0.60), followed by simulated homologous breakpoints (*R*^2^ = 0.45), and meiotic DSB (*R*^2^ = 0.39). This suggests a predominant role of spontaneous DSBs in the origin of germline NAHR events. We generated a multiple linear regression model using the 3 variables together and observed improved prediction (adjusted *R*^2^ = 0.66; *P* < 2.2 × 10^−16^). Result obtained from regression by random forest was consistent with linear regression (Supplementary Figure S8). Further detailed analysis revealed that the majority of bins with discordant predictions (56/79, 70%) had lower predicted than observed values (Figure 2B). We, thus, concluded that the absence of DSB and homology correctly predicted the absence of NAHR; however, these variables were less precise for predicting a higher number of NAHR events. Although, A1 comprises only ∼12% of the 4064 bins, most, i.e. 26 (∼49%) of these 53 bins were A1, suggesting a role for open chromatin in further increasing the NAHR count.

To study the role of open chromatin, we performed an advanced simulation of NAHR breakpoints, in which the simulated homologous breakpoints set was further normalized based on length and compartment distribution of real NAHR deletions (see Materials and Methods). Compartment normalized density of chromatin interaction between breakpoints of different origins was compared (Figure 2C). Such normalization eliminated biases related to uneven distribution of SVs of different origins across compartments having overall different chromatin interaction strength (Supplementary Figure S9). Indeed, density distributions for random points, simulated NAHR breakpoints, and NHrepl breakpoints (that are not expected to show any association with chromatin as occurring during replication with no defined chromatin organization) matched perfectly (Figure 2C). Breakpoints of NAHR deletions had significantly stronger chromatin interaction than that of NHEJ (Kolmogorov–Smirnov (KS) test, *P* = 4.9 × 10^−5^) and that of simulated set (KS test, *P* = 3.9 × 10^−5^). This observation points to a preference for NAHR deletion ends to have higher Hi-C contact, which also implies physical proximity. Furthermore, NAHR breakpoints with only surrounding spontaneous DSBs had stronger interaction (KS test, *P* = 0.008) than NAHR breakpoints with only surrounding meiotic DSBs (Figure 2D), consistent with the role of interacting distal DNA fragments in the creation of NAHR events caused by spontaneous DSBs.

In the absence of sister chromatid and homologous chromosome, HDR relies on non-allelic homologous sequence templates for DSB repair. The homologous sequences often belong to a distant genomic region and any 3D physical proximity with DSB site will benefit HDR. Indeed, experiments in haploid budding yeast showed that physical proximity of the donor/template to be rate-limiting step for HDR of DSBs (33). Accordingly, errors in HDR would also reflect this physical proximity. Thus, we concluded that NAHR around spontaneous DSBs likely arise as errors of HDR. Association of NAHR breakpoints with H3K79me2 (Supplementary Figure S7), might point toward regional bias for 53BP1 promoted error-free HDR. Although, counter intuitive, probably indicative of NAHR deletion being rare outcome of otherwise efficient HDR.

### Repair related SVs are associated with chromatin accessibility

**Figure 3.**
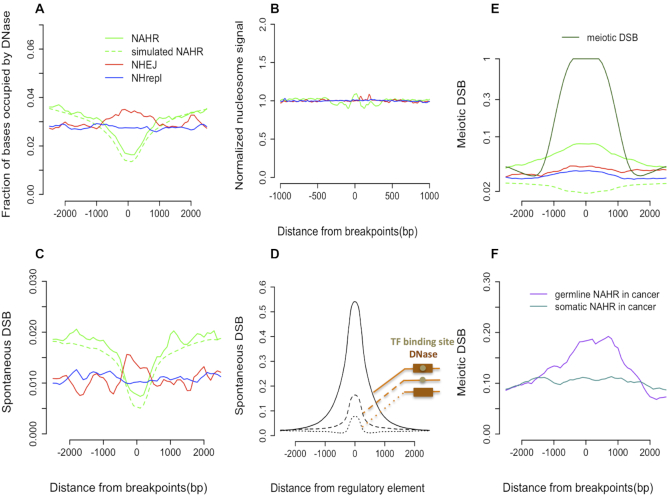
(**A**) NHEJ breakpoints are enriched with DNase I hypersensitive sites, unlike NHrepl breakpoints. (**B**) No enrichment/depletion of normalized nucleosome signal around 1 kb of repair related deletions. (**C**) Aggregations for spontaneous DSB peaks are similar to those for DNase sites. (**D**) Aggregation of spontaneous DSBs around regulatory elements. The largest enrichment is observed for transcription factor (TF) binding site inside DNase sites. (**E**) Meiotic DSBs are enriched only around NAHR breakpoints; however, the enrichment is not as high as would be expected if all NAHR events were of meiotic origin. (**F**) Enrichment of meiotic DSBs was observed around germline NAHR but not around somatic NAHR in cancer.

SV breakpoint regions were further analyzed to understand the effect of local chromatin accessibility on certain mechanisms of DNA repair on a smaller, sub-compartment scale. DNase I hypersensitive sites indicate local chromatin accessibility and spontaneous DSBs correlate strongly with DNase sites (Supplementary Figure S10) as also observed previously (15). The aggregated DNase signal shows depletion around NAHR breakpoints (Figure 3A). However, since the set of simulated NAHR follows exact same trajectory as the real one, the depletion is likely caused by compromised read mapping from DNase experiments in repeats around breakpoints. However, DNase content in the surrounding region (1–2.5 kb) of NAHR is higher than that around non-homologous deletions. This is consistent with preferential location of NAHR breakpoints in A type compartments, which in turn are enriched with DNase sites. The same observations and interpretations are valid for associations with spontaneous DSBs (15) (Figure 3C). These observations further demonstrate the importance of studying SVs in terms of large compartments that is not biased by local sequence properties.

Unlike NAHR breakpoints, non-homologous breakpoints are not enriched with repeats. While replication based non-homologous breakpoints do not show any preference for DNase sites, DNase content is enriched around NHEJ breakpoints (*t*-test, *P* < 0.01) (Figure 3A). The latter is unlikely to be a feature of accessible chromatin, as the same enrichment is not observed for nucleosome free linker regions (Figure 3B). Furthermore, we observed enrichment of spontaneous DSB signal around NHEJ breakpoints (*t*-test, *P* < 0.01) but not NHrepl ones (Figure 3C). As NHEJ deletions are byproduct of DSB repair machinery, enrichment of DNase sites around NHEJ is likely due to correlation of spontaneous DSBs and DNase sites (Supplementary Figure S10). Moreover, we found that DNase sites, which are also occupied with transcription factor binding sites, have much stronger association with spontaneous DSBs than either DNase sites or binding sites alone (Figure 3D). These observations indicate the role of co-localization of transcription factor binding sites and DNase sites in the genesis of SVs, as has been observed for SNVs previously (11).

Contrary to DNase sites and spontaneous DSBs (mean length 408.7 bp), discovery of meiotic DSBs (mean length 1417.2 bp) were unlikely to be affected by genomic repeats. This is evident by the difference in aggregated signal for the real and simulated NAHR which verifies that the observation is not confounded by repeat contents and other features associated with NAHR (Figure 3E). In agreement with the classical model of NAHR, in which meiosis is a prerequisite, aggregation of meiotic DSBs around NAHR breakpoints shows significant enrichment (*t*-test, *P* < 0.01), whereas for non-homologous deletions this is not the case (Figure 3E). However, if all NAHRs were of meiotic origin, then they would be expected to be in the same location of meiotic DSB peaks and the enrichment of meiotic DSB around breakpoints would be expected to be higher, i.e. the normalized aggregated signal should be 1. The reduction in this enrichment can be rationalized if most NAHR deletions are not of meiotic origin but rather are the result of repair errors of spontaneous DSBs. In support of this suggestion, analysis of germline and somatic NAHR deletions from cancer genomes (10) revealed enrichment of meiotic DSBs around breakpoints of germline deletions (*t*-test, *P* < 0.01) whereas such enrichment is not observed around somatic breakpoints (Figure 3F). By comparing observed and predicted (when all breakpoints are assumed to be of meiotic origin) aggregation of meiotic DSB signal around NAHR breakpoints, we estimated that approximately 10% of NAHR deletions arise from meiotic DSBs (see Materials and Methods).

### Length of retrotransposed sequences, nucleosome periodicity and chromatin accessibility

**Figure 4.**
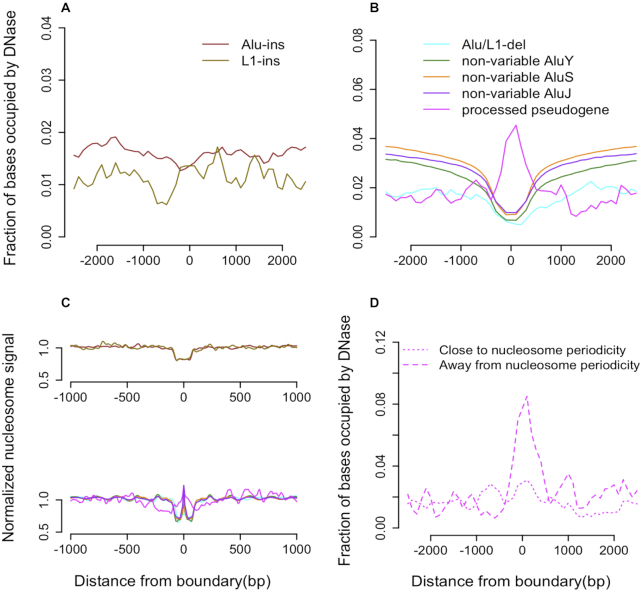
(**A**) No enrichment/depletion of DNase sites is observed around retrotransposons discovered as insertions. (**B**) Depletion of DNase sites is observed around breakpoints of deleted retrotransposons as well as non-variable Alu elements, suggesting that the effect is due to compromised read mapping around repeats. Insertion sites of processed pseudogenes are enriched with DNase sites. (**C**) Retrotransposon insertion sites are depleted with nucleosome signal. TEI, non-variable Alu, and processed pseudogenes also support depletion of the nucleosome signal around breakpoint. The peak at the center of the X-axis (lower panel) represents the normalized mean nucleosome signal within element boundaries. (**D**) DNase signal is enriched only around pseudogenes where length is away from nucleosome periodicity (∼150 bp).

The signal from DNase I hypersensitive sites was neither depleted nor enriched around Alu and L1 insertions (Figure 4A). On the contrary, we observed depletion of signal around retrotransposon breakpoints detected as deletions (*t*-test, *P* < 0.01) (Figure 4B). As detected insertions and deletions of retrotransposons are all ancestral insertions, the difference in the aggregated signal is likely explained by the repetitive sequence content of transposable elements. Specifically, the repeat content can confound DNase site detection around transposable element deletions, which are present in the reference genome, and have no confounding effect on detecting DNase sites around retrotransposon insertions, which are absent in the reference genome. Indeed, a similar depletion of non-variable Alu elements supports this explanation. Thus, we found no preference for retrotransposable elements to insert into accessible chromatin. However, as was previously known (34), we observed a preference for insertions into the linker regions between nucleosomes (*t*-test, *P* < 0.01) (Figure 4C). Both DNase sites and linker regions are nucleosome free, and the preference for insertion into the latter can be explained by the former being occupied by transcription factors or being under strong negative selection. Additional analysis suggests, that transcription factor binding sites are unlikely to have a significant role in this because we did not observe any difference in aggregation of DNase sites that are free of binding site (data not shown).

Transposase also modulates insertion of processed pseudogenes. The DNase signal was enriched (t-test, *P* < 0.01) (Figure 4B) around boundaries of young processed pseudogenes (>95% identity with parent gene), with nucleosome signal, while being similar to that of retrotransposons, exhibiting difference (Figure 4C). Compared with transposons, pseudogenes can vary in length. We further classified pseudogenes based on whether their length is close or away from nucleosome periodicity (∼150 bp). We found enrichment of DNase signal around pseudogenes whose length was away from the periodicity whereas this enrichment was absent for the other class (Figure 4D). This observation suggests a role of pseudogenes in creating new DNase sites (nucleosome free region) due to excess, i.e. more than whole number of periods, DNA sequence. We didn’t observe any association between nucleosome periodicity and the length of repair-related deletions (Supplementary Figure S11).

## DISCUSSION

In this study, we described analysis of germline SVs of different origins in the context of epigenomic properties. Although, epigenome vary in different cell types, that does not affect conclusions from our analysis (Supplementary Figures S12–S14). We observed a biased distribution of SVs across genome compartments, epigenetic states, and functional annotations, likely portraying the complex interplay of differential mechanistic biases, chromosome confirmation, DNA accessibility, and natural selection. Because of these factors, SVs of different origin affect genome in different manners.

For instance, various roles for pseudogenes have been proposed, including providing mRNA stability for the parent gene, gene silencing via generation of siRNA, and sponging for miRNA and thereby regulating the parent gene (35). Based on our results, we propose that processed pseudogenes can create new DNase sites and thus generate new regulatory elements. Such elements are the result of inserting sequence (of a pseudogene) that deviates from the whole number of nucleosome periods and breaks nucleosome periodicity. Selection pressure (or perhaps a mechanism) to maintain the periodicity results in a residual (over whole nucleosome periods) sequence that remains open and accessible.

NAHR deletions are more likely to disrupt topologically associated domain boundaries. These domain boundaries serve as insulators that prohibit any inter-domain interaction. It has been shown that disruption of domain boundaries may generate disease conditions through ectopic interactions (30). Such events were reported for ensemble of diseases such as cancer (36), neurological (37) and congenital diseases (38). Additionally, NAHR deletions are more likely to affect transcribed regions, particularly those with weak transcription. Although there are fewer deletions of NAHR origin as compared to non-homologous mechanisms, they could have comparable or stronger functional consequences. Given that read-pair and split-read approaches for SV discovery are underpowered to find events from NAHR, studies ignoring read depth approach are potentially underpowered in identifying functionally relevant SVs.

The predominant origin of NAHR deletions has been a subject of debate. The classical theory states that NAHR happen as errors during directed recombination in meiosis (39). Additionally, NAHR could be the result of an error in HDR that is initiated by a spontaneous DSB. Our analysis indicates that majority of NAHR in germline originate from the latter. This conclusion is based on several observations: (i) association of NAHR with recombination hotspots was rather weak; (ii) spontaneous DSBs, a prerequisite for repair, strongly correlated with NAHR occurrence across genome compartments and was the best predictor of NAHR density; 3) NAHR breakpoints had stronger physical interactions than breakpoints from other mechanisms. Direct observation of association of NAHR with DSBs, as we showed, is unlikely to be observed because of the limitations of existing experimental approaches to identify DSBs in repeats flanking NAHR breakpoints. The predominant occurrence of NAHR deletions from errors in DNA repair implies that most of them happen either in germ cells prior to meiosis (mosaic in parent and *de novo* in children) or during early development (mosaic in children) (40–43).

Different from NAHR and counter intuitively, NHEJ breakpoints were not associated with spontaneous DSBs on a large compartment scale. This observation is likely attributable to a preference for germline cells to use high fidelity HDR for repair of DSBs, particularly in A compartments, where a template sequence could be taken from a nearby homologous region, while in B compartments, deficient in local homologous templates, HDR is superseded by NHEJ. This explanation is further supported by molecular evidences of HDR as a preferable choice in active chromatin in Aymard *et al.* (44). Together, these results can also explain observed non-random distribution of repair choices during CRISPR–Cas9 mediated targeted DSBs (45) and predict biased outcome of HDR mediated genome engineering (46,47) based on genomic region of interest. Our conclusion about the choice of DNA repair mechanism is also consistent with NHEJ breakpoints having lesser physical interaction than random points and NAHR breakpoints. Specifically, reliance on homology by HDR would favor utilization of templates from more frequently interacting region, while mutually exclusive nature of HDR and NHEJ would bias breakpoints from the latter mechanism toward less interacting regions. However, since we observed association of NHEJ with spontaneous DSBs on a smaller (kb) scale, we conclude that breakpoints of deletions related to errors in DNA repair (i.e. both from NHEJ and NAHR) are primarily associated with sites of DSBs, while regional sequence features, histone modifications, and interplay between the repair mechanisms lead to differential association of breakpoints from each mechanism to compartments and functional states. Similar observations were previously reported for SNV burden in both germline and soma (48–50). In contrast to NAHR and NHEJ, and as a positive control, for replication based non-homologous deletions (that happen mostly due to collapse of replication fork), we observed no relation with DSBs.

Open chromatin is intrinsically more prone to DSBs than closed chromatin, the consequence of widespread DNase sites in the former. But interestingly transcription factor occupied DNase sites were associated with DSBs, while transcription factor free DNase sites were not. This difference may reflect ‘a hard to reach’ effect that has been described for somatic SNVs in cancers (11), such that occupancy of DNase sites with transcription factors hampers efficient repair of DSBs. Alternatively, it is possible that binding of transcription factors enables instability in the surrounding region, thereby introducing DSBs (51–53). More intriguingly, as A compartments encompass the most functionally relevant chromatin, which simultaneously is most vulnerable to DSBs, maintaining their stability is likely to be paramount task for a cell and organism in order to reduce errors when passing genome information to next generation and to maintain viability of somatic cells, thereby ensuring viability of an individual (54). Our simulation of NAHR breakpoints suggests that availability of a local template for HDR in A compartments is primarily driven by the abundance and enrichment of old, fixed copies of Alu elements in that compartment. Contrary to that, young, variable Alu insertions are enriched in B compartments, revealing disproportional distributions of old and young Alus across the genome (Supplementary Figure S15). On a local, nucleosome-size scale, both old and young Alus exhibited similar associations, indicating no difference in their mechanism of retrotransposition.

Although, Alu elements comprise ∼10% of human genome, less is known about reason of their high abundance (55). Previous works suggested various regulatory roles of Alu elements (56–59). Here, we hypothesize that the human genome (and likely other primates) have evolved such that A compartments are enriched for homologous sequences to promote repair by HDR, which is a higher fidelity mechanism than NHEJ (Supplementary Figure S16). In this model, old Alu insertions in open chromatin were preferentially retained to promote the use of HDR for genome repair. Although, HDR is not error-free, studies of several organisms have shown that crossover of double-Holliday junctions (the event generating a deletion) is rare in non-meiotic cell (54,60,61). The preferential use of HDR led to lowering the overall rate of incorrect repairs, while the frequency NAHR (i.e. mistakes of HDR) increased simply due to more frequent use of the HDR. Existing theories of genome instability due to Alu elements (23,62,63) can be naturally synergized with our hypothesis. The suggested genome instability due to retrotransposons, Alus in particular, is the ‘price’ of a more stable genome overall. Furthermore, the prevalence of same directional Alus in the human genome (62,64) suggests selection pressure acting on Alus during their expansion, consistent with our hypothesis. Once the optimal stability was achieved, the newer insertions are disruptive in open chromatin, leading to the enrichment of recent insertions in B compartments.

Where (i.e. in which cell type) and why nature selected to maintain higher stability is an open question. Oocytes pass genetic information to next generation; consequently, maintaining their genome stability is of the highest importance. This suggestion is consistent with existing reports that most germline NAHR deletions are of maternal origin (65). Repeat expansion in those cells could particularly boost repairs by HDR in the absence of sister chromatid and homologous chromosome.

## Supplementary Material

Supplementary Data
