# Supplementary material for: Chromatin organization modulates the origin of heritable structural variations in human genome
Source: Nucleic Acids Res. 2019 Feb 18;47(6):2766–77. doi: 10.1093/nar/gkz103 (PMC6451188; doi:10.1093/nar/gkz103)
Supplement: Supplementary Data [file gkz103_supplemental_file.pdf]

Supplementary Materials

Chromatin organization modulates the origin of heritable structural variations in human genome

Tanmoy Roychowdhury[1] and Alexej Abyzov[1,*]

[1]Mayo Clinic, Department of Health Sciences Research, Center for Individualized Medicine, Rochester, MN 55905

[*]Send correspondence to abyzov.alexej@mayo.edu

Table S1: Description of 15 states generated by chromHMM using 5 histone marks (Epigenome Roadmap)

**Abbreviation**	**Description**
TssA	Active TSS
TssAFlnk	Flanking Active TSS
TxFlnk	Transcription at gene 5' and 3'
Tx	Strong Transcription
TxWk	Weak Transcription
EnhG	Genic Enhancers
Enh	Enhancers
ZNF/Rpts	ZNF genes and Repeats
Het	Heterochromatin
TssBiv	Bivalent/Poised TSS
BiVFlnk	Flanking Bivalent TSS/Enh
EnhBiv	Bivalent Enhancers
ReprPC	Repressed Polycomb
PeprPCWK	Weak Repressed Polycomb
Quies	Quiescent/Low

Table S2: Gender specific recombination rates in different compartments

Compartment	Mean Recombination rate in Male	Mean Recombination rate in Female
A1	0.82429	1.055923
A2	0.91751	1.004883
B1	1.56823	1.597996
B2	1.22927	1.007922
B3	0.85665	0.802174

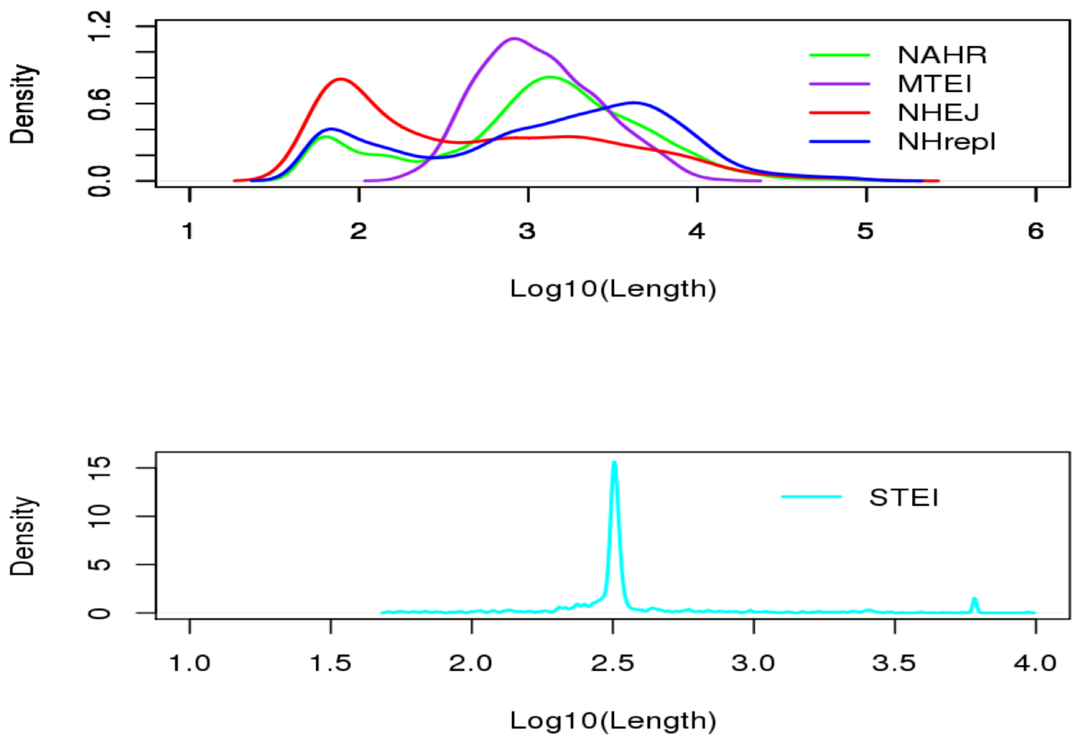

Figure S1: Length distribution of different SV types.

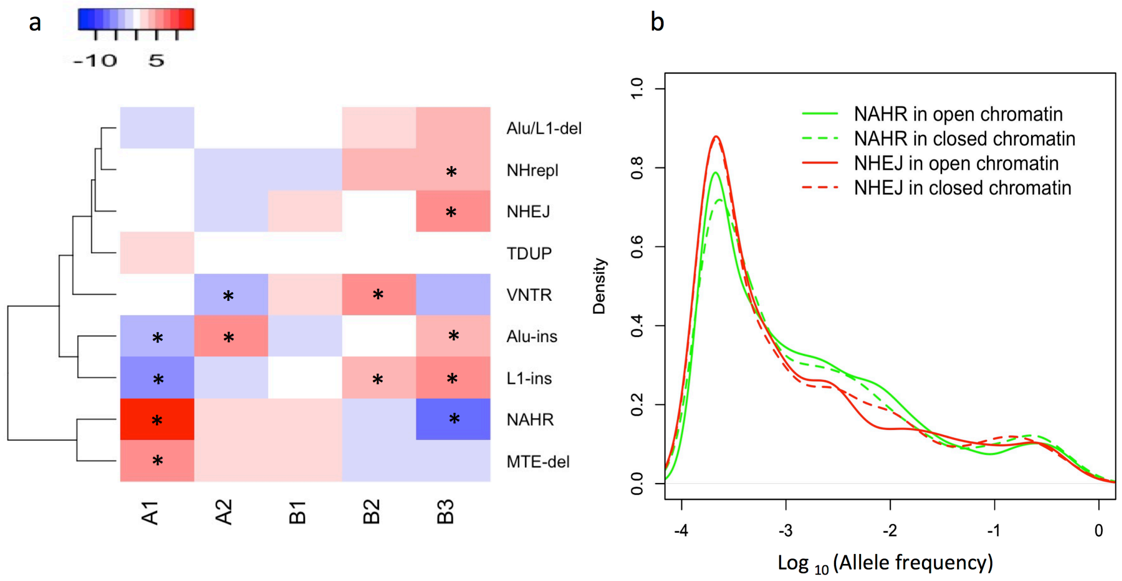

Figure S2: (a) Clustering of SV classes based on their enrichments by Z-score in 5 compartments, identified from Hi-C studies. In figure 1a, tandem duplications have lowest number of events (357). In this figure, we used only 357 randomly drawn events from each SV class. (b) Comparison between distributions of minor allele frequency in open vs. closed chromatin. Differences were not significant in either NAHR (KS test; P-value=0.26) or NHEJ (KS test; P-value=0.21).

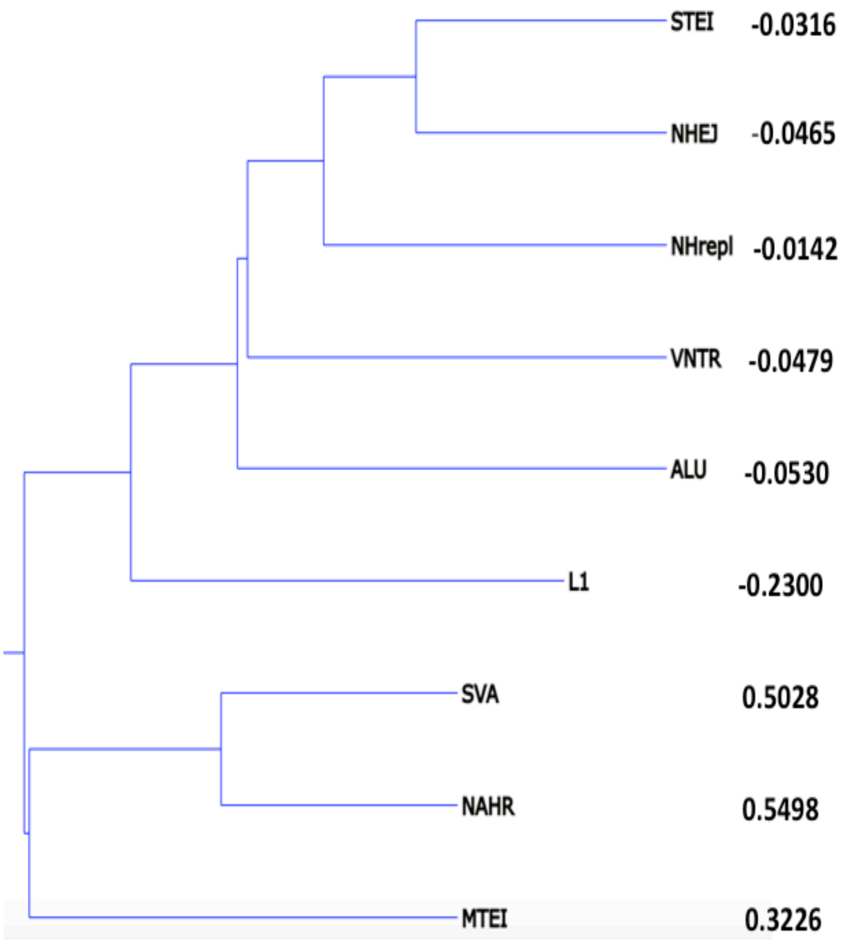

Figure S3: Clustering of SV types based on replication time (mean replication time on right).

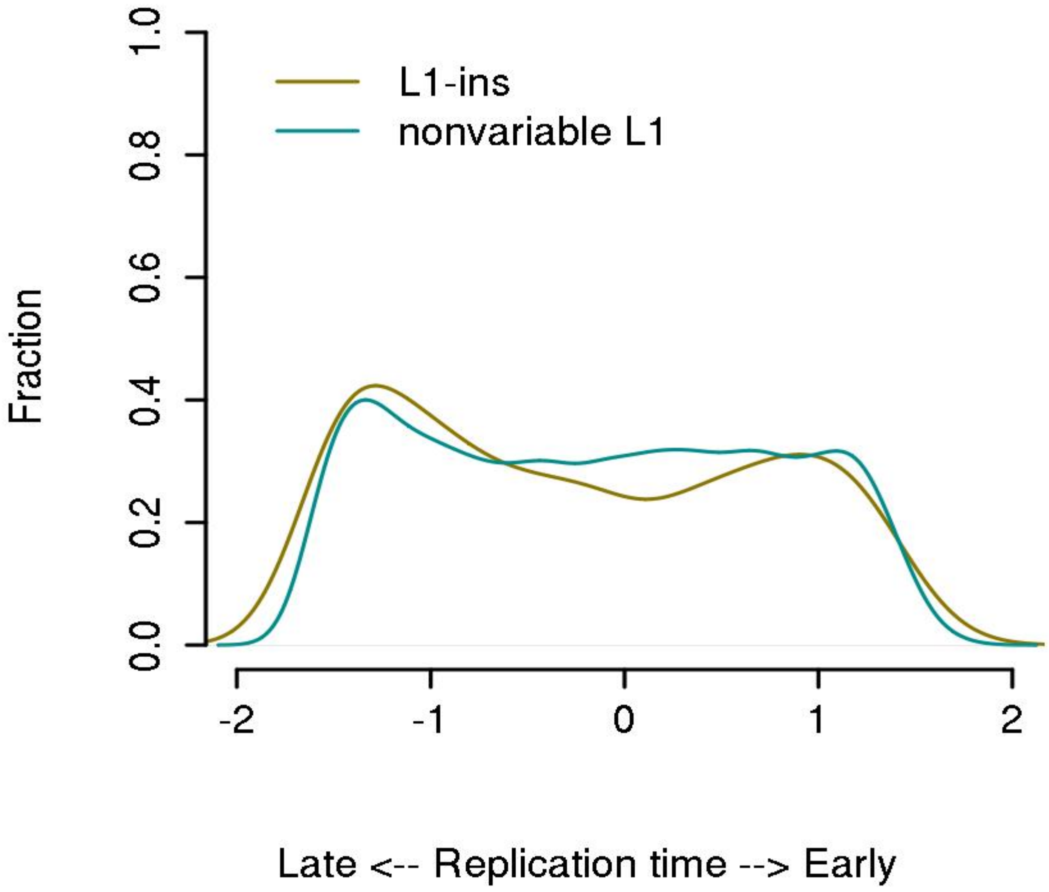

Figure S4: Normalized distribution of replication timing for L1 insertion and nonvariable L1 sites.

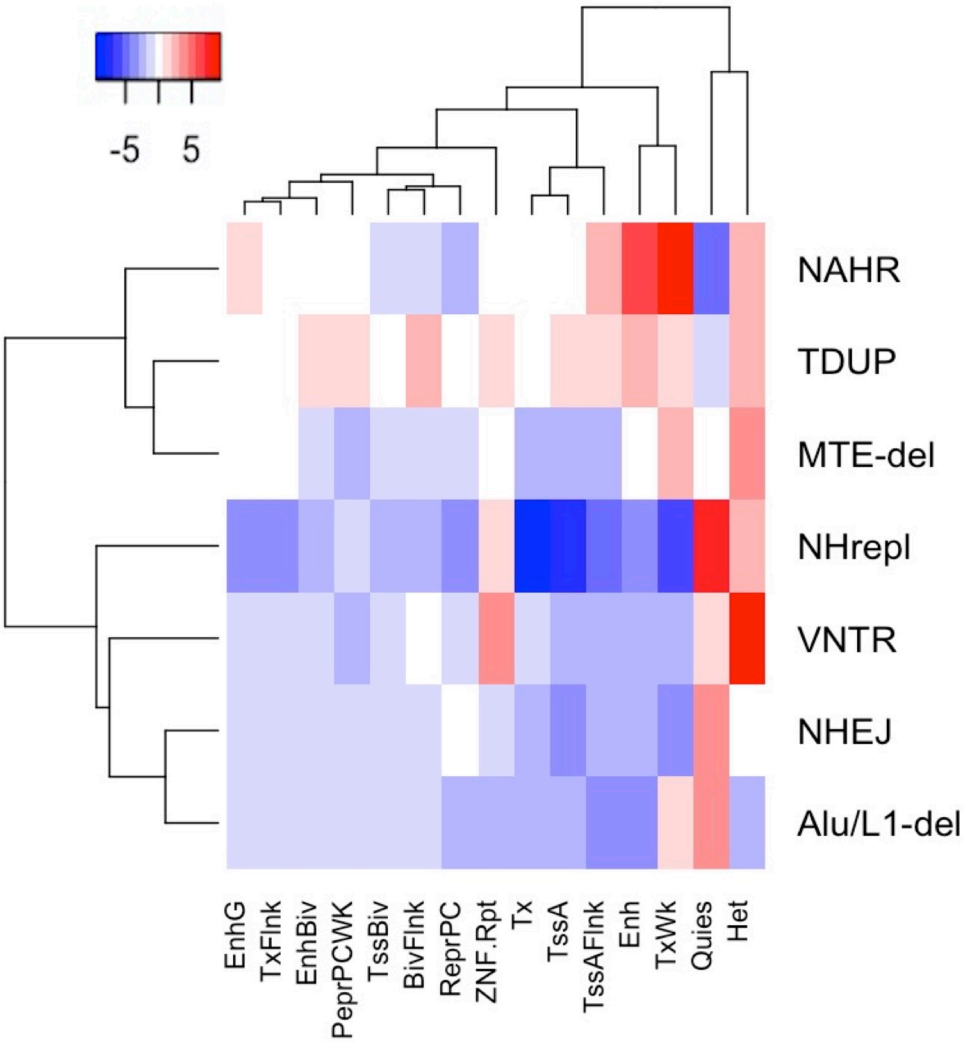

Figure S5: Clustering of SV classes and functional states based on deleted/duplicated region in the states. The states were inferred from chromHMM segmentation of histone marks.

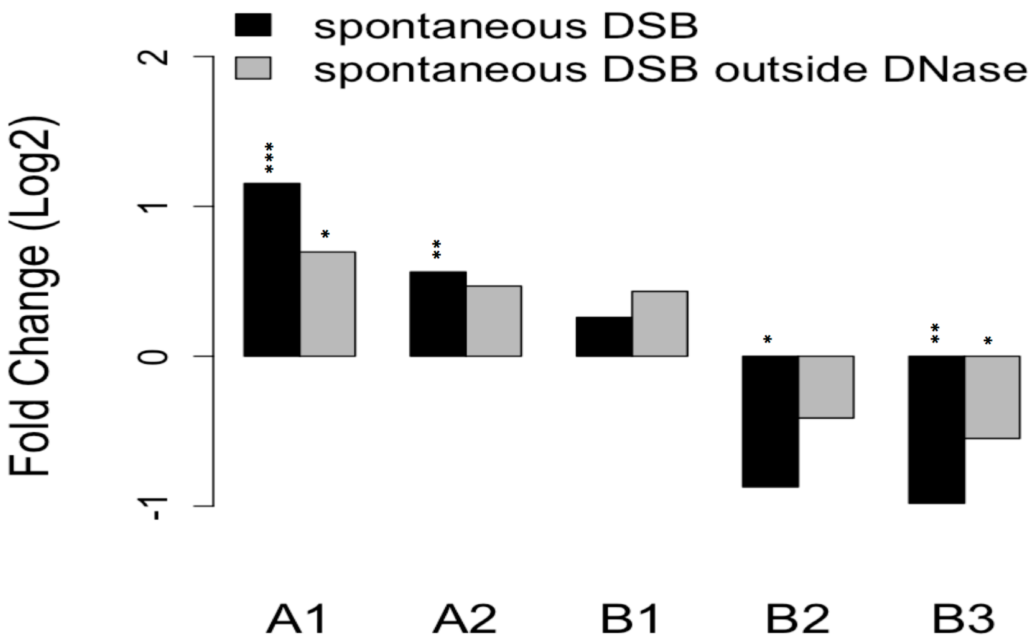

Figure S6: Fold enrichment (Log_2 scale) of all spontaneous DSBs and those outside DNase regions in five compartments as compared to uniform distribution based on compartment lengths. P-values were calculated using chi-square test (* means p-value < 0.05, ** means p-value < 0.005, *** means p-value < 0.0005).

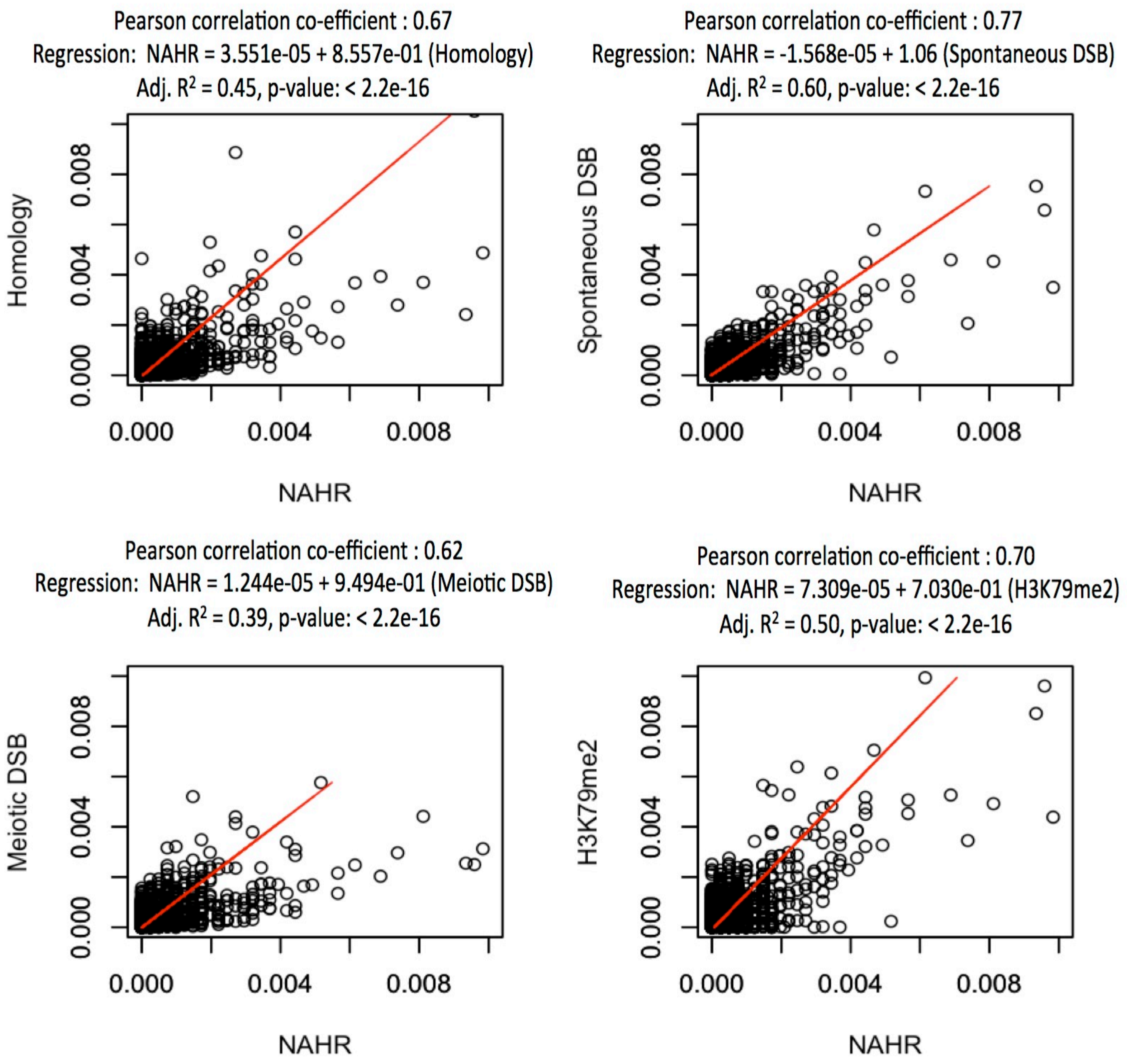

Figure S7: Linear regression (Y~X) using 4 independent variables separately. Y = Fraction of NAHR deletion in each bin (compartment). X = Fraction of simulated homologous breakpoints/ spontaneous DSB peak/ meiotic DSB peak / H3K79me2 peak in each bin (compartment).

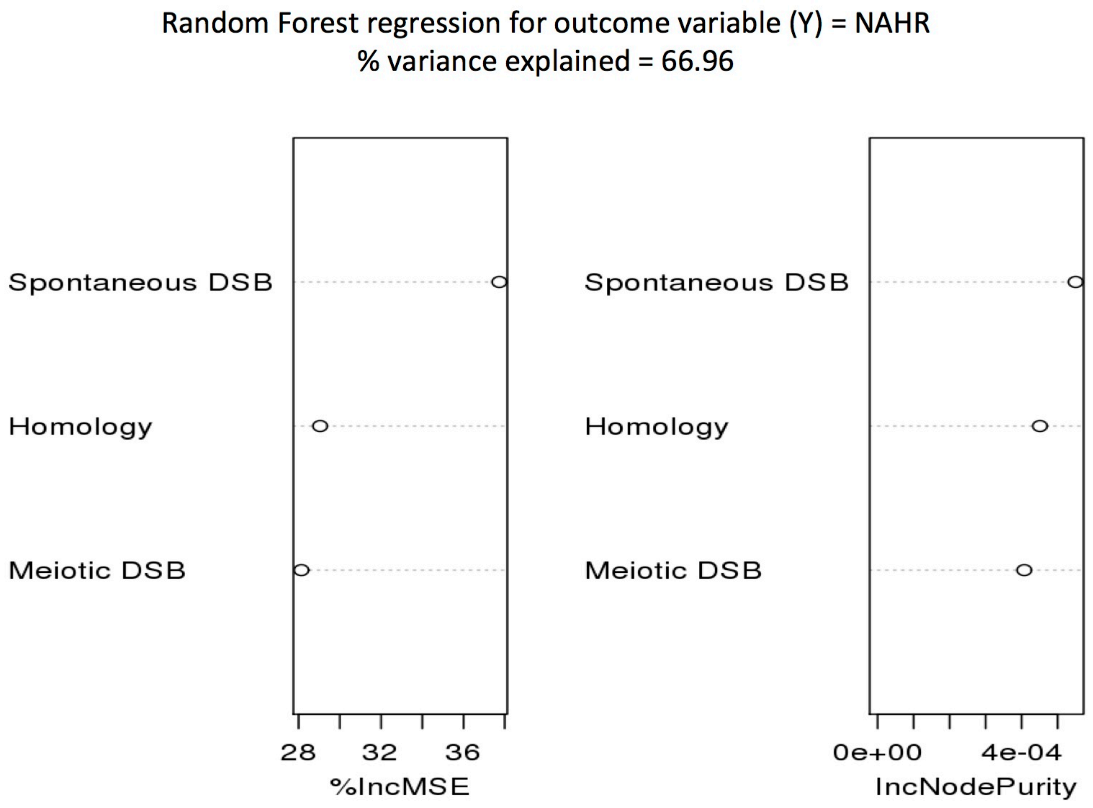

Figure S8: Variable importance (%IncMSE and IncNodePurity) based on random forest regression with NAHR as outcome variable. Both metrics suggest that spontaneous DSBs are best predictor of NAHR followed by homology and meiotic DSB.

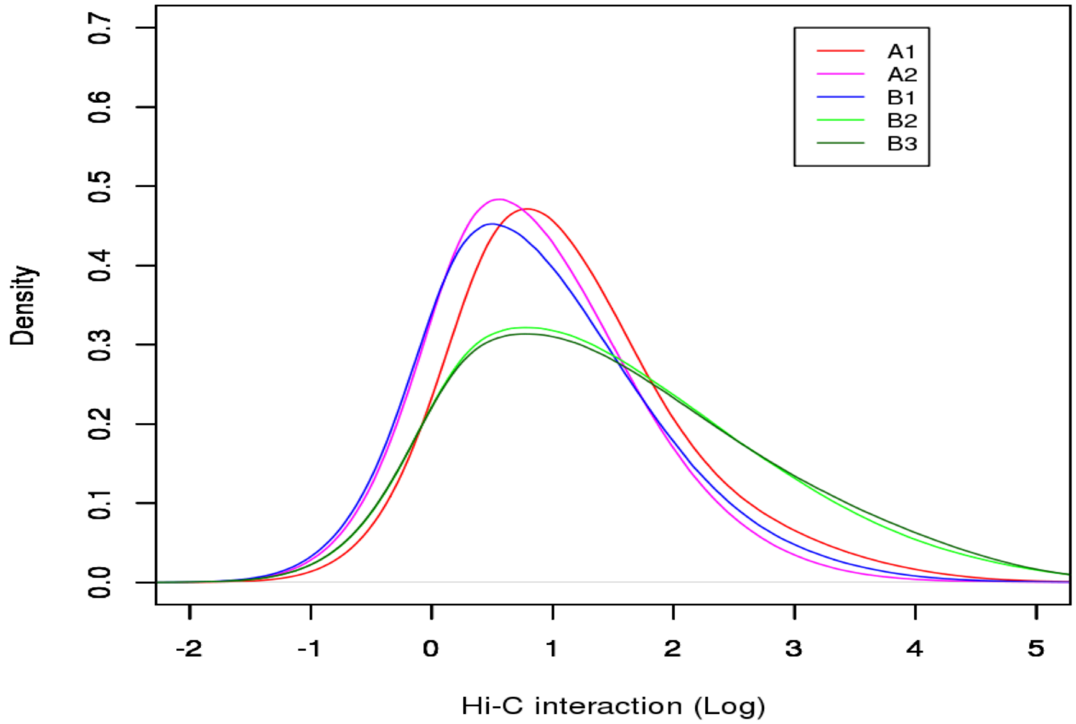

Figure S9: Hi-C interaction among random points (in same compartment).

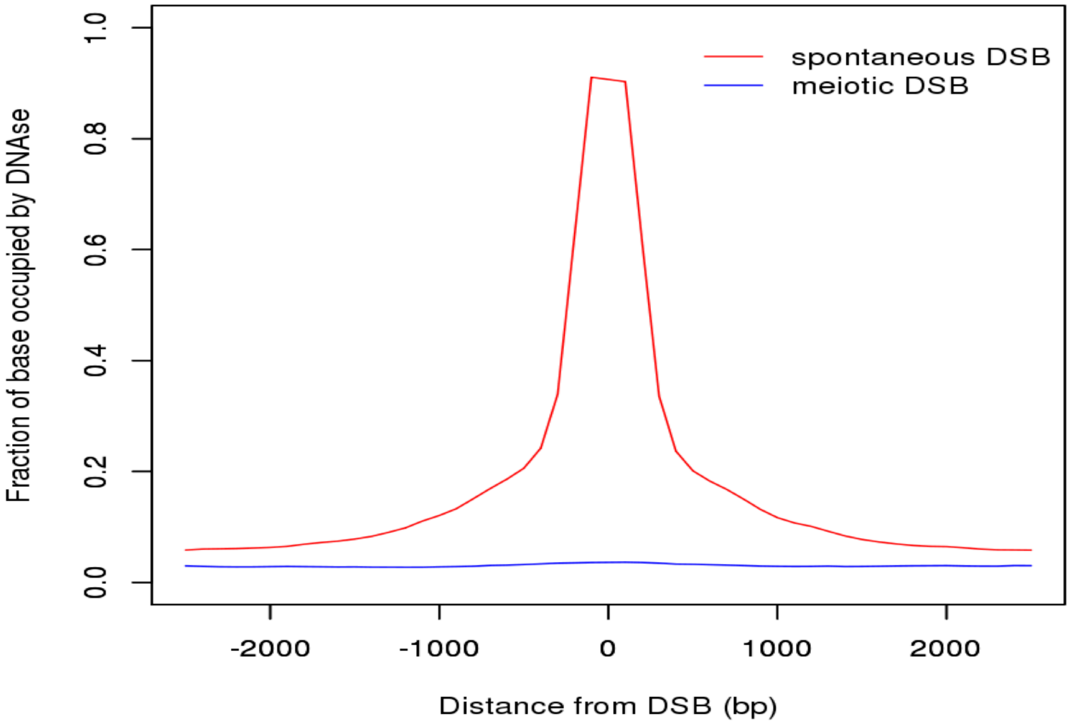

Figure S10: Aggregation of DNase sites around DSBs.

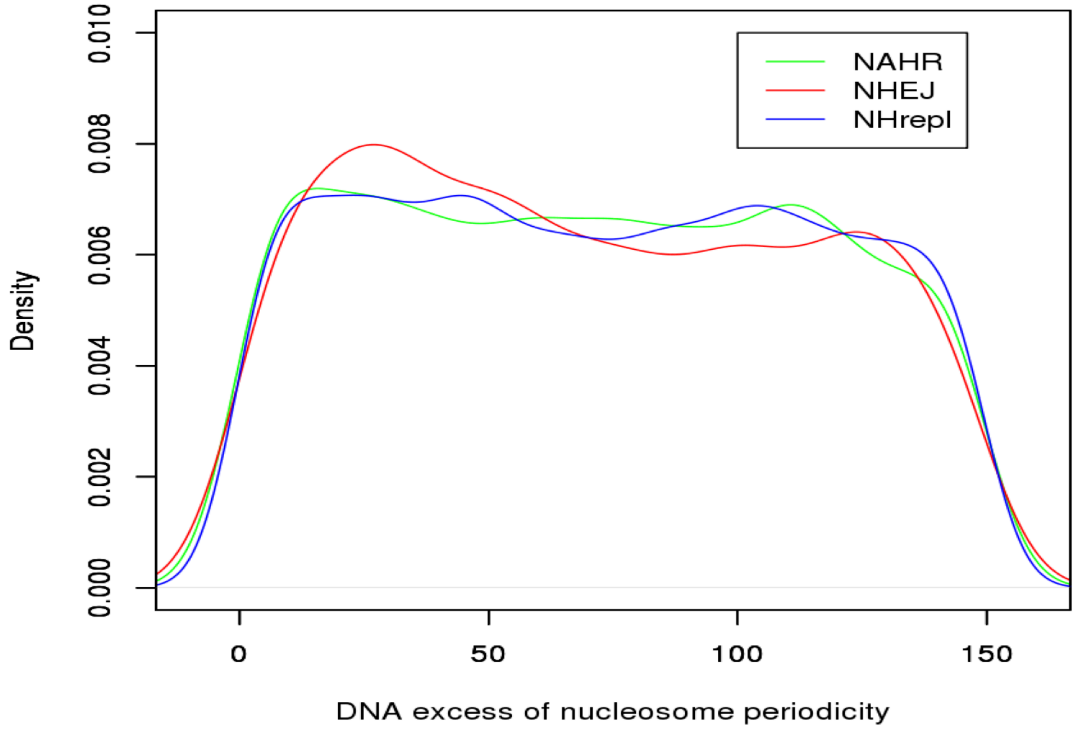

Figure S11: Excess length over the whole number of nucleosome periods for repair related deletions. Only deletions longer than 150 bp were considered.

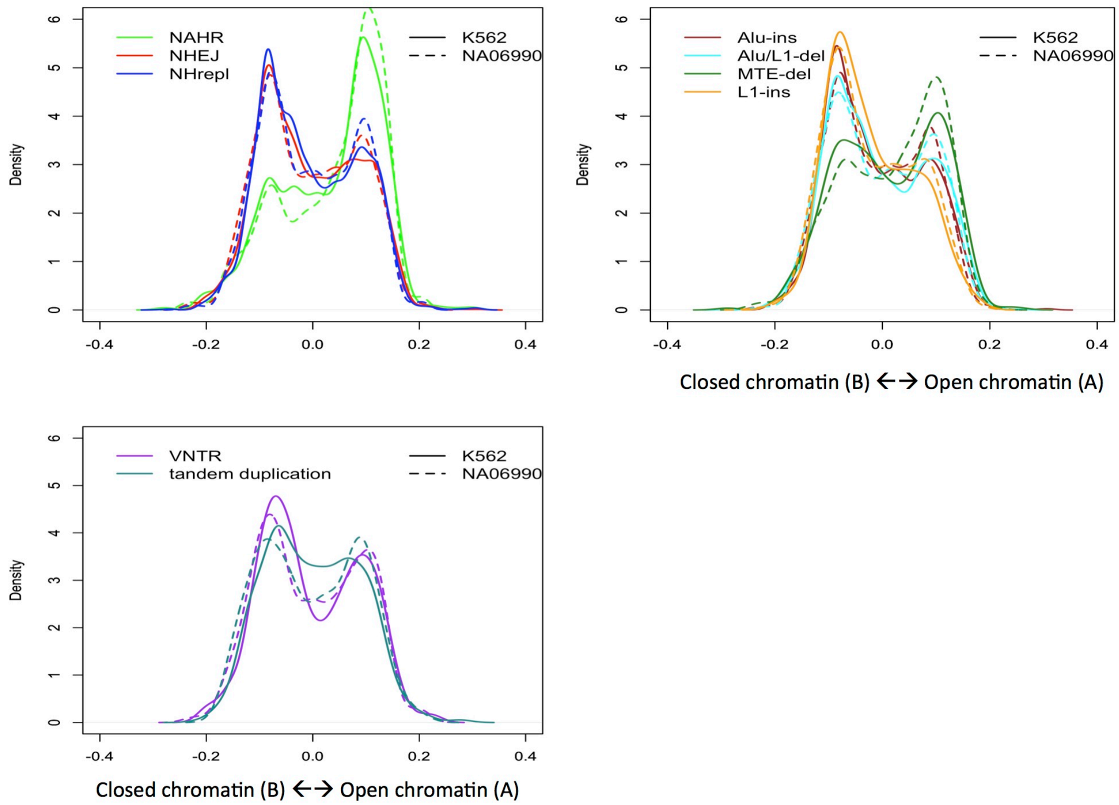

Figure S12: Distribution of Hi-C eigen vectors of bins with SV breakpoints from different origin. Hi-C data from K562 (cancer) and NA06990 (lymphoblastoid) give consistent results with observations from figure 1a.

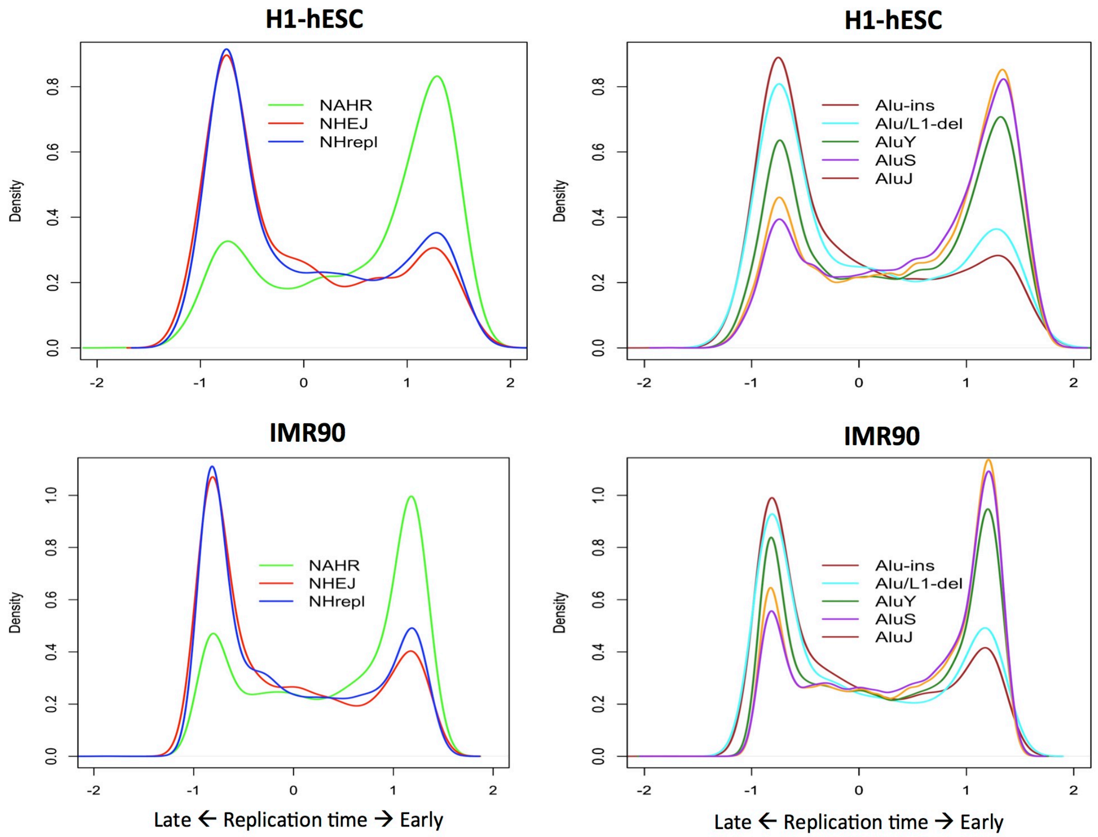

Figure S13: Normalized distribution of replication timing for SV breakpoints from different origin using data from H1-hESC (embryonic stem cell) and IMR90 (fetal lung). Results are consistent with figure 1b, S3.

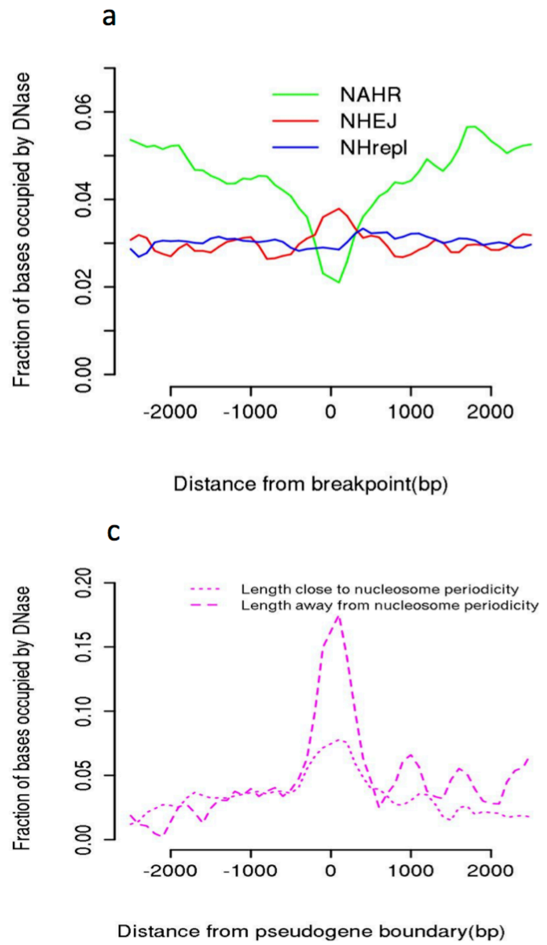

Figure S14: Plots similar to main figures using DNase I hypersensitive sites from H1-hESC. a) same as Fig. 3a b) same as Fig. 4a-b c) same as Fig. 4d

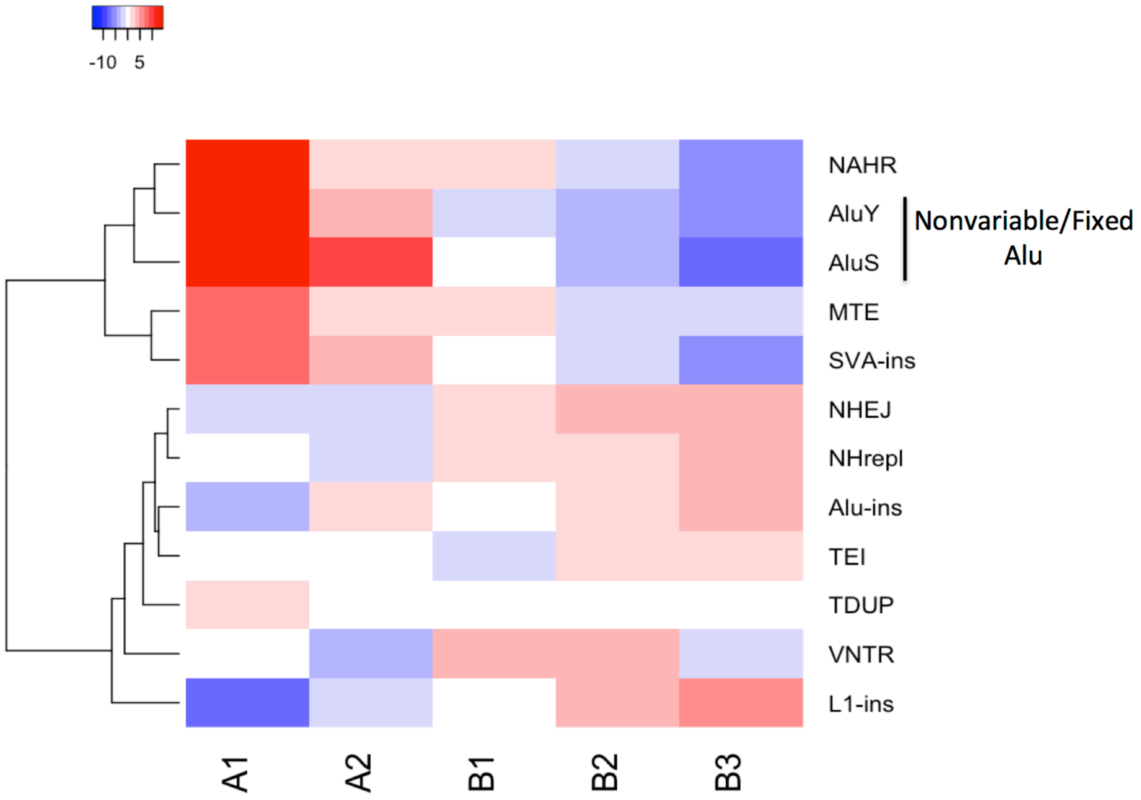

Figure S15: Clustering of SV classes and non-variable Alu elements based on their distribution in five compartments identified from Hi-C studies.

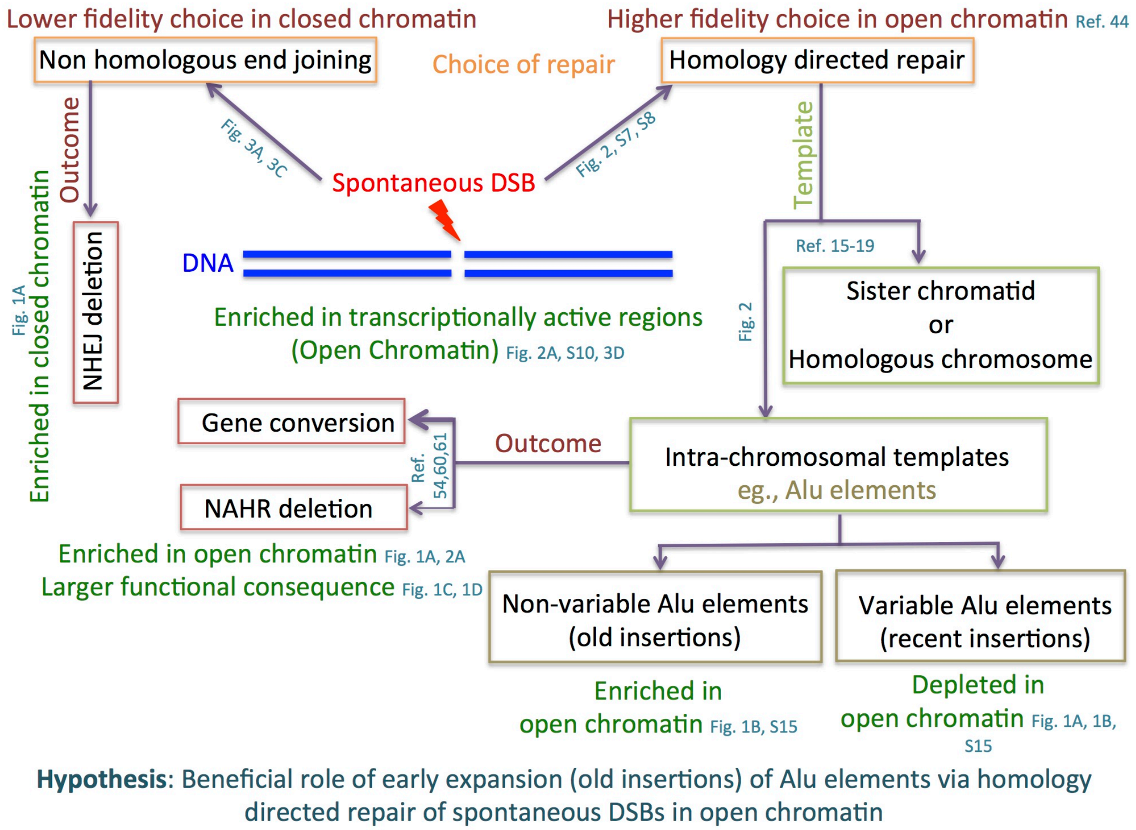

Figure S16: A summary of our major observations.
